# Novel deep learning for multi-class classification of Alzheimer’s in disability using MRI datasets

**DOI:** 10.3389/fbinf.2025.1567219

**Published:** 2025-08-20

**Authors:** Sumaiya Binte Shahid, Maleeha Kaikaus, Md. Hasanul Kabir, Mohammad Abu Yousuf, A. K. M. Azad, A. S. Al-Moisheer, Naif Alotaibi, Salem A. Alyami, Touhid Bhuiyan, Mohammad Ali Moni

**Affiliations:** ^1^ Institute of Information Technology, Jahangirnagar University, Savar, Dhaka, Bangladesh; ^2^ Department of Mathematics and Statistics, College of Science, Imam Mohammad Ibn Saud Islamic University (IMSIU), Riyadh, Saudi Arabia; ^3^ School of IT, Washington University of Science and Technology, Alexandria, VA, United States; ^4^ Artificial Intelligence and Cyber Futures Institute, Charles Stuart University, Bathurst, NSW, Australia; ^5^ AI and Digital Health Technology, Rural Health Research Institute, Charles Sturt University, Orange, NSW, Australia; ^6^ Health Sciences Research Center (HSRC), Deanship of Scientific Research, Imam Mohammad Ibn Saud Islamic University (IMSIU), Riyadh, Saudi Arabia

**Keywords:** disability research, Alzheimer’s disease (AD), dementia, deep learning, magnetic resonance imaging (MRI), convolutional neural network (CNN), transfer learning

## Abstract

**Introduction:**

Alzheimer’s disease (AD) is one of the most common neurodegenerative disabilities that often leads to memory loss, confusion, difficulty in language and trouble with motor coordination. Although several machine learning (ML) and deep learning (DL) algorithms have been utilized to identify Alzheimer’s disease (AD) from MRI scans, precise classification of AD categories remains challenging as neighbouring categories share common features.

**Methods:**

This study proposes transfer learning-based methods for extracting features from MRI scans for multi-class classification of different AD categories. Four transfer learning-based feature extractors, namely, ResNet152V2, VGG16, InceptionV3, and MobileNet have been employed on two publicly available datasets (i.e., ADNI and OASIS) and a Merged dataset combining ADNI and OASIS, each having four categories: Moderate Demented (MoD), Mild Demented (MD), Very Mild Demented (VMD), and Non Demented (ND).

**Results:**

Results suggest the Modified ResNet152V2 as the optimal feature extractor among the four transfer learning methods. Next, by utilizing the modified ResNet152V2 as a feature extractor, a Convolutional Neural Network based model, namely, the ‘IncepRes’, is proposed by fusing the Inception and ResNet architectures for multiclass classification of AD categories. The results indicate that our proposed model achieved a standard accuracy of 96.96%, 98.35% and 97.13% for ADNI, OASIS, and Merged datasets, respectively, outperforming other competing DL structures.

**Discussion:**

We hope that our proposed framework may automate the precise classifications of various AD categories, and thereby can offer the prompt management and treatment of cognitive and functional impairments associated with AD.

## 1 Introduction

Alzheimer’s disease (AD) is a progressive neurological disorder that slowly destroys a person’s memory, thinking skills, and general consciousness, ultimately affecting their ability to performc daily activities (Raza et al., 2023). Early detection of Mild Cognitive Impairment (MCI) can prevent or slow the progression of MCI to Alzheimer’s disease (AD) (El-Assy et al., 2024). Normal healthy control (NC), mild cognitive impairment (MCI), and Alzheimer’s disease are the stages that precede dementia. The patient can avoid developing severe AD by receiving prompt diagnosis and therapy. According to information obtained from the World Alzheimer’s Report, more than 55 million people have been diagnosed with this disease, and this number is expected to reach 78 million by 2030 (Faisal and Kwon, 2022).

The current AD diagnostic process usually includes radiological as well as clinical evaluations. In clinical settings, doctors use behavioural assessments, patient histories, and cognitive tests like the Mini-Mental State Examination (MMSE) and the Alzheimer’s Disease Assessment Scale-Cognitive Subscale (ADAS- Cog). In terms of radiology, Magnetic Resonance Imaging (MRI) is highly regarded for its capacity to identify anatomical abnormalities in the brain related to Alzheimer’s disease (AD) (Ghosh et al., 2023). Recent research has revealed notable performance in the multimodality data-based identification and classification of AD. The patient’s clinical records, Positron Emission Tomography (PET), Computed Tomography (CT), Magnetic Resonance Imaging (MRI), X-rays and other imaging modalities are included in the multimodal data (Nawaz et al., 2020).

MRI is a very helpful and secure method of monitoring the changes in the brain caused by AD. It is essential in the clinical system and plays a key role in identifying the disease. Various machine learning and deep learning algorithms have been applied to identify Alzheimer’s disease (AD) from MRI scans. However, adjacent stages on the CDR (Clinical Dementia Rating) spectrum often present with overlapping neuroimaging characteristics, making fine-grained classification challenging. Based on the impressive results of deep learning and Convolutional Neural Networks (CNN) in image classification, this work analyzes the efficacy of employing CNNs (Convolutional Neural Networks) for extracting characteristics from MRI scans to identify Alzheimer’s disease. Recognizing images like a human can be challenging for a machine, but it becomes much simpler with the support of computer vision using a deep neural network (Bangyal et al., 2022). Most studies on Alzheimer’s disease (AD) using Neural Networks worked with 2D images, while those using 3D images mainly focused on binary classifications (Hon et al., 2017; Zhang et al., 2021; Ullanat et al., 2021).

This demonstration shows how brain MRI scans are used to improve Alzheimer’s Disease (AD) classification. It highlights the use of neural networks to analyze brain anatomy and summarizes findings using publicly available datasets. A more advanced model is developed which helps for identifying different stages of Alzheimer’s Disease.

The main contribution of our research is outlined as follows:

• Our proposed IncepRes model, which is the combination of Inception modules with ResNet, leverages the strength of classification performance.
• The study confirms that the combination of modified ResNet152V2 as feature extractor and IncepRes as classifier outperforms seven other combinations utilizing four modified pre-trained models for feature extraction and two classifiers for classification.
• By conducting comprehensive experiments, the model has been validated compared to the existing models and its efficacy has been demonstrated.

The remainder of the paper is divided into the following sections: A review of existing works is done in Section 2. In Section 3, a thorough explanation of all the materials and methods, including the feature extraction, selection, and classification procedure is explained. The result, analysis, and all other validation and verification techniques are covered in Section 4. Finally, Section 5, 6 presents the discussion and conclusion.

## 2 Literature review

Several studies have developed various categorization algorithms for AD diagnosis and detection systems. A summary of recent research on AD diagnostic and detection systems using traditional ML and DL techniques is presented in this section.

Machine learning algorithms can automatically improve their performance and accuracy over time as they are exposed to more data (Bangyal et al., 2023). Different machine learning models have been developed to perform Alzheimer’s disease detection on MRI images. M. Sudharsan and G. Thailambal suggested AD detection on 214 subjects from the ADNI dataset using Informative Vector Machine (IVM), Random Extreme Learning Machine (RELM), and Support Vector Machine (SVM). RELM outperformed the other two classifiers in terms of accuracy and feature selection methods for differentiating between AD, MCI, and HC (Sudharsan and Thailambal, 2023). A five-stage machine learning pipeline with integrated data transformation and feature selection strategies was presented in Khan and Zubair (2022) for automated classification of AD using Mini-Mental State Examination (MMSE), Clinical Dementia Rating (CDR), and Average Score Final (ASF) scores. Bucholc et al. developed a novel hybrid prognostic machine learning framework that integrated unsupervised learning with supervised models, leading to improved accuracy in predicting the progression of mild cognitive impairment (MCI) to dementia (Bucholc et al., 2023). However, a balanced dataset of high-quality MRI scans from AD patients and healthy controls is necessary for accurate AD categorization using a machine learning algorithm.

CNN has become a popular approach for the diagnosis of AD. Shamrat et al. proposed a model named AlzheimerNet which outperformed traditional methods for classifying the stages of Alzheimer’s disease, as validated by a two-tailed Wilcoxon signed-rank test with a significance of 0.05 (Shamrat et al., 2023). A CNN-based model was proposed by Fazal Ur Rehman Faisal et al. on sMRI brain images from ADNI datasets, which was used to mix features from various layers to hierarchically transform the magnetic resonance imaging images into more compact high-level features (Faisal and Kwon, 2022). The YOLOv3 object detection algorithm was trained in Koga et al. (2022) to detect five tau lesion types using 2,522 images from 10 cases of AD, Progressive Supranuclear Palsy (PSP), and Corticobasal Degeneration (CBD). Xiuli Bi et al. introduced a fully unsupervised deep learning model for diagnosing AD. The following two components make up the suggested method. First, they used an unsupervised CNN called PCANet to learn features from MRI scans. Secondly, for the final diagnosis of AD, they turned to the unsupervised classification approach based on k-means (Bi et al., 2020). An end-to-end framework is built by Helaly et al. for early detection of Alzheimer’s disease using deep learning approaches, including simple CNN architectures and transfer learning, which attained 97% accuracy (Helaly et al., 2022). CNN was utilised by Bae et al. (2020) to develop a classification algorithm for AD based on 2D segments of T1-weighted MRI scans containing AD-sensitive brain regions from two distinct groups of diverse cultural and racial identities.

One study implemented and compared various deep models, including 2D and 3D CNNs and RNNs. The 3D voxel-based method achieved 96.88% accuracy, 100% sensitivity, and 94.12% specificity Ebrahimi et al. (2021b). In Kang et al. (2021), a novel EL CNN system was implemented, which combined 11 of the top validation accuracy 2D slice-level models from three CNNs. Ahmad Waleed Salehi et al. developed a CNN-based model where CNNs were comprised of neurons with biases and weights tailored to the various objects in a picture (Salehi et al., 2020). Their proposed model only focused on a coronal view of the MRI image. Besides, CNN requires a significant amount of labeled data for classifying AD.

Artificial neural networks (ANN) play a significant role in medical imaging and healthcare applications. The improved bat algorithm (IBA) proposed in Bangyal et al. (2019) demonstrated superior performance over traditional backpropagation and other optimization techniques, making it a promising approach for complex medical image analysis. Amir Ebrahimi et al. utilized a variety of deep sequence-based models (Ebrahimi et al., 2021a) to diagnose AD. The developed sequence-based models included the temporal convolutional network (TCN) and numerous RNN types. However, the authors found the issue of overfitting in this particular scenario.

To distinguish AD from HC sMR pictures, a customized Inception-ResNet model was created by Sreelakshmi Shaji et al. Accuracy of this paper is 69% (Shaji et al., 2021). By using images segmented by the brain’s grey matter (GM), Noman Raza et al. explored the categorization and segmentation of MRI of Alzheimer’s disease using the ideas of transfer learning and CNN customization (Raza et al., 2023). Sreeja Sasidharan Rajeswari et al. implemented a model where the efficacy of the transfer learning method was investigated in depth by fine-tuning the deeper layers of TL models, including VGG-19, VGG-16, Resnet- 50, and Xception (Rajeswari and Nair, 2021). In one study (Hon et al., 2017), the fully-connected layer was retrained using MRI scans, and the scientists used two separate models, VGG16 and InceptionV4, softmax classifier, and sMRI biomarkers, for the classification of AD. However, this paper could only show binary classification.

The proposed DenseNet-121 model in Hazarika et al. (2022) achieved notable results with an average performance rate of 88.78%, and by incorporating depth-wise convolution, the accuracy further improved to 90.22%, highlighting its potential in AD classification. To classify AD, M. Tanveer et al. presented the “Deep Transfer Ensemble (DTE),” an ensemble of deep neural networks that is effective to compute and independent of DL architecture (Tanveer et al., 2021). To identify and categorize the various stages of AD, Nagarathna C R et al. experimented and compared the performance of a CNN deep learning model and a hybrid model that combined VGG19 and additional layers (Nagarathna and Kusuma, 2021). In research work, the researcher appeared with CNN-based models to identify Alzheimer’s diseases from MRI images with three different classifiers named Softmax, RF, and SVM. The rate of accuracy is 99% and 96% for ADNI and MIRIAD, respectively (AlSaeed and Omar, 2022). The authors of this paper provided the information on whether a person had AD or not but could not do classification.

To summarize the limitations of existing models, it is noticed that achieving better performance is easy in binary classification, but it is difficult to achieve better accuracy in multiclass classification. Besides, due to the complexity of DL models and 3D neuroimaging biomarkers, it is challenging to demonstrate which specific features have been extracted and to control how those characteristics influence the inference and relative prominence of different features. Moreover, pre-processing steps for manual feature extraction can be error-prone. According to comprehensive assessment, several trials used only one dataset. In response to these challenges, we have focused on improving our IncepRes model by enhancing its accuracy in classifying different stages of dementia, improving the extraction of key features from MRI data, and optimising the integration of these processes. We have also rigorously validated the model, demonstrating that it has outperformed seven other combinations. These improvements have addressed significant limitations in existing methods and have made the classification process more effective and reliable.

Table 1 presents an overview of recent important research on detecting Alzheimer’s disease. It includes a summary of different methods used in these studies, such as transfer learning, deep learning, and traditional machine learning techniques. This table summarizes key findings and approaches from these studies, showing progress in Alzheimer’s detection and diagnosis.

**TABLE 1:** Recent research using deep learning, machine learning, and transfer learning to detect and classify Alzheimer’s Disease.

Method	Author	Contribution	Limitations
ML	Sudharsan and Thailambal (2023)	Compared IVM, RELM, and SVM. RELM achieved higher accuracy and feature selection for AD from MCI and HC	Lower accuracy in multiclass classification
ML	Khan and Zubair (2022)	Selected features including age, ADAS13, MMSE, and MRI hippocampal and midtemporal lobe measurements	Limited data led to lower accuracy in cMCI
DL	Bi et al. (2020)	Used unsupervised CNN for feature extraction and prediction	Validated on small datasets; K-means not fine-tuned
DL	Kang et al. (2021)	Proposed an ensemble CNN model with multi-slice integration	Lacked optimized slice selection strategy
DL	Salehi et al. (2020)	Combined 2D and 3D MRI for feature extraction	Focused only on the coronal view of MRI
TL	Hon et al. (2017)	Pre-trained CNN on large image dataset to extract PET features	Limited to binary classification
DL + TL	Raza et al. (2023)	Classified 2D GM slices into four AD classes using SPM12	Negative transfer risk from problem similarity
DL + TL	AlSaeed and Omar (2022)	Proposed CNN-based feature extraction surpassing handcrafted methods	Only diagnosed presence of AD

## 3 Materials and methods

**FIGURE 1:**
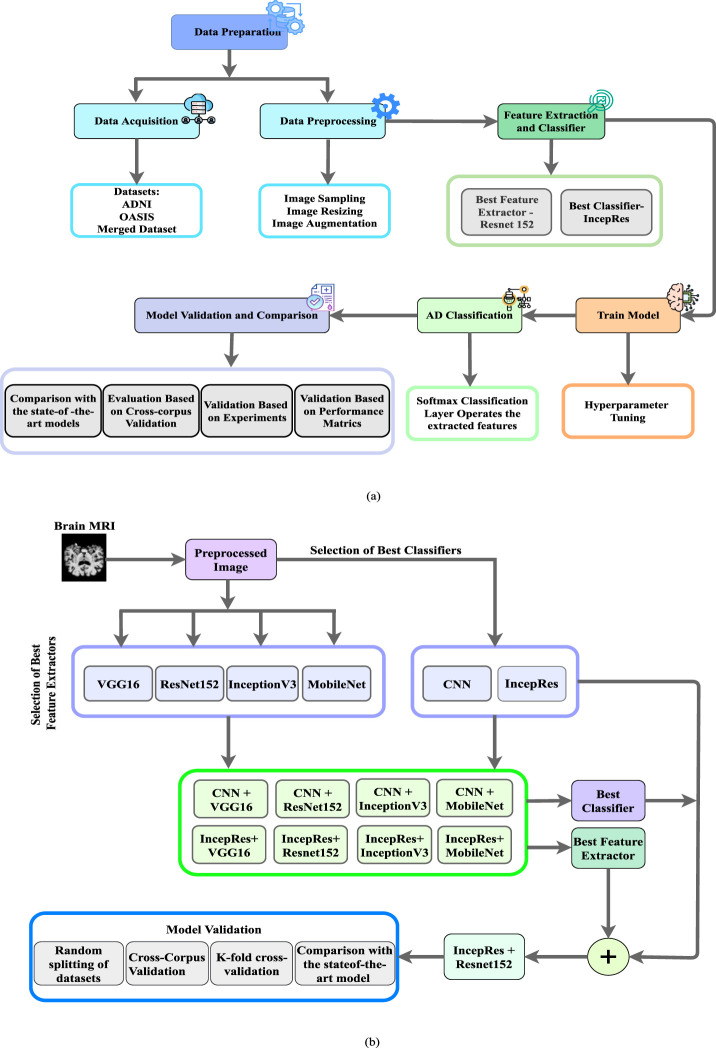
**(a)** Workflow of the proposed method to depict the processes of preprocessing, feature extraction, classification, and model validation, **(b)** Schematic Diagram of our Proposed Method.

The essential abstract plan for carrying out research is known as research planning. The basic task and the necessary work action are both included in research planning. A firm grasp of the study plan might aid the researcher in realizing the bounds and restrictions of the work. A structural outline has been created containing the steps to implement our research. Figure 1a depicts the detailed workflow of the proposed method such as preprocessing, feature extraction, classification, and model validation while Figure 1b gives us a representation of Schematic Diagram of our proposed method to detect the stages of Alzheimer’s. We have performed pre-processing on the obtained MRI data to prepare it for analysis. The preprocessed data is then passed into a feature extractor. The outcomes of a range of models have been assessed to find the best one for feature extraction. The training and testing datasets were carefully balanced by the severity of the target pathology, including Non-Demented (ND), Very Mild Demented (VMD), Mild Demented (MD), and Moderate Demented (MoD) cases, to ensure a balanced representation across all stages of Alzheimer’s disease. After extracting the features, it is fed to the optimal classifier. Lastly, the proposed model has been validated by considering several features and experiments to verify its performance. We have utilized 7-fold cross-validation to rigorously assess the model’s adaptability. This method has ensured that each subset of data is used for both training and testing, with no overlap between the two, enhancing the model’s robustness.

### 3.1 Dataset

In this study, we have utilized two publicly available MRI datasets, ADNI ADNI (2003), OASIS Marcus et al. (2007), and a dataset combining ADNI and OASIS referred to as Dataset 1, Dataset 2 and Merged Dataset respectively. Both datasets include comprehensive subject assessments, with cognitive impairment levels determined by the Clinical Dementia Rating (CDR) score. CDR values of 0, 0.5, 1, and 2 correspond to cognitive categories of Non-Demented (ND), Very Mild Demented (VMD), Mild Demented (MD), and Moderate Demented (MoD), representing increasing dementia severity (Marcus et al., 2007). These categorizations ensure consistency across the datasets, allowing for standardized multi-class classification of Alzheimer’s disease stages.

#### 3.1.1 Dataset 1

Alzheimer’s Disease Neuroimaging Initiative (ADNI) ADNI (2003) was created by multicenter longitudinal investigations. Some North American national institutes, including NIA and NIBIB, launched ADNI as a 5-year collaboration program in 2004. We have utilized baseline 3T T1-weighted MRI scans from ADNI dataset. T1-weighted axial MRI images are obtained which consist of 1,224 images in total. Among these images, there are 171 images classified as MoD, 580 as ND, 233 as MD, and 240 as VMD.

#### 3.1.2 Dataset 2

The Open Access Series of Imaging Studies (OASIS) (Marcus et al., 2007) project aims to provide brain neuroimaging datasets to the community of scientists without any charge. In our research, we have used neuroimaging data from MRI sessions performed on 414 patients (314 persons without dementia, 70 subjects in the Very Mild stage of dementia, 28 subjects having Mild Dementia and two subjects with Moderate Dementia) from OASIS-2. This dataset consists of 216 participants between the ages of 18 and 59 and 198 between the ages of 60 and 96. An approximately equal number of male and female subjects comprise each group. Among the elderly participants, 98 have a CDR score of zero, indicating no dementia, while 100 have a CDR score above zero (70 with CDR = 0.5, 28 with CDR = 1, and 2 with CDR = 2), indicating very mild to moderate Alzheimer’s disease.

#### 3.1.3 Merged dataset

To enhance the model’s robustness and assess its generalizability, we have created a merged dataset by combining MRI images from both the ADNI and OASIS datasets. The combined dataset consists of a total of 1,638 images across four classification categories: Non-Demented (ND), Very Mild Demented (VMD), Mild Demented (MD), and Moderate Demented (MoD). This merged dataset contains 894 images classified as ND, 310 as VMD, 261 as MD, and 173 as MoD.

### 3.2 Data preprocessing

**FIGURE 2:**
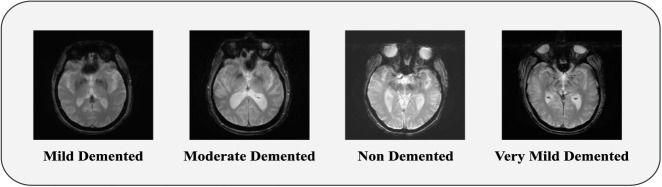
MRI sample of Mild Demented, Moderate Demented, Non Demented and Very Mild Demented.

The first and main step in creating a machine learning model is creating and making the data suitable. The data needed for the model is not always clean and formatted. We have to process the data from the raw and noisy conditions. This process is called the data pre-processing (AlSaeed and Omar, 2022). Data acquisition is done to collect data from different sources for pre-processing. Two publicly available datasets, ADNI (2003), Marcus et al. (2007) and a Merged dateset containing four types of brain MRI, are utilized to train our model. Samples of MRI images from each type are shown in Figure 2. We have done image resizing, rescaling and augmentation as our pre-processing step.

#### 3.2.1 Image resizing

Resizing an image alters its dimensions, which usually affects its file size and can also reduce the quality. The most frequent reason for resizing images is to minimize the size of large files to make them suitable for any application. The dimension of our images before preprocessing was 256 × 256. Transfer learning inputs must be suitable for the learned model Ahamed et al. (2021). For this reason, a pixel resolution of 224 × 224 is applied to images for resizing.

#### 3.2.2 Image rescaling

Image rescaling alters the data range and the data points’ location. The ratio of the width to height must be preserved while rescaling. In our image rescaling step, the scaling value is set as 1/.255, and the offset is fixed to −1.

#### 3.2.3 Data augmentation

Augmentation of Data is a mechanism that uses existing data to create modified copies of datasets, which are then used to increase the training dataset artificially (Faruqui et al., 2021). It reduces the overfitting issues. Our employed datasets have an obvious class imbalance issue. In Dataset 1, the ND class has almost twice as much data as all the other classes combined. Whereas, Dataset 2 demonstrates imbalances across all classes. To address this issue, we have proposed a methodology based on transfer learning. Initially, it performs real-time data augmentation and utilizes these augmented data points as inputs for modified transfer learning models. We have applied six different augmentation procedures (Rotation, Shear, Zoom, Horizontal flip, Brightness range set, and Contrast set) on our datasets. The state-of-the-art effectiveness can be improved by data augmentation (Arafa et al., 2022). Table 2 incorporates columns indicating the count of data samples before and after augmentation, in addition to presenting the distribution of samples across the Training, Validation, and Testing sets for each dataset category. We have fine-tuned our pre-trained models for our imbalanced datasets, including merged dataset. Through fine-tuning, overfitting of the majority class is prevented while allowing the model to learn from the entire dataset.

**TABLE 2:** Total number of data samples in different datasets, including augmented data and splits for training, validation, and testing.

Dataset	Category	Sample number	Augmented data	Training	Validation	Testing
1	MD	233	1,314	1,065	118	131
	MoD	171	949	769	85	95
	ND	580	2,952	2,391	266	295
	VD	240	1,349	1,093	121	135
	Total	**1,224**	**6,564**	**5,318**	**590**	**656**
2	MD	28	166	134	15	17
	MoD	2	12	10	1	1
	ND	314	1718	1,391	155	172
	VMD	70	414	336	37	41
	Total	**414**	**2,310**	**1871**	**208**	**231**
Merged	MD	261	1,305	1,044	131	138
	MoD	173	865	692	87	89
	ND	894	4,470	3,576	447	468
	VMD	310	1,550	1,240	135	145
	Total	**1,638**	**8,190**	**6,552**	**800**	**840**

### 3.3 Feature extractors and classifier

**FIGURE 3:**
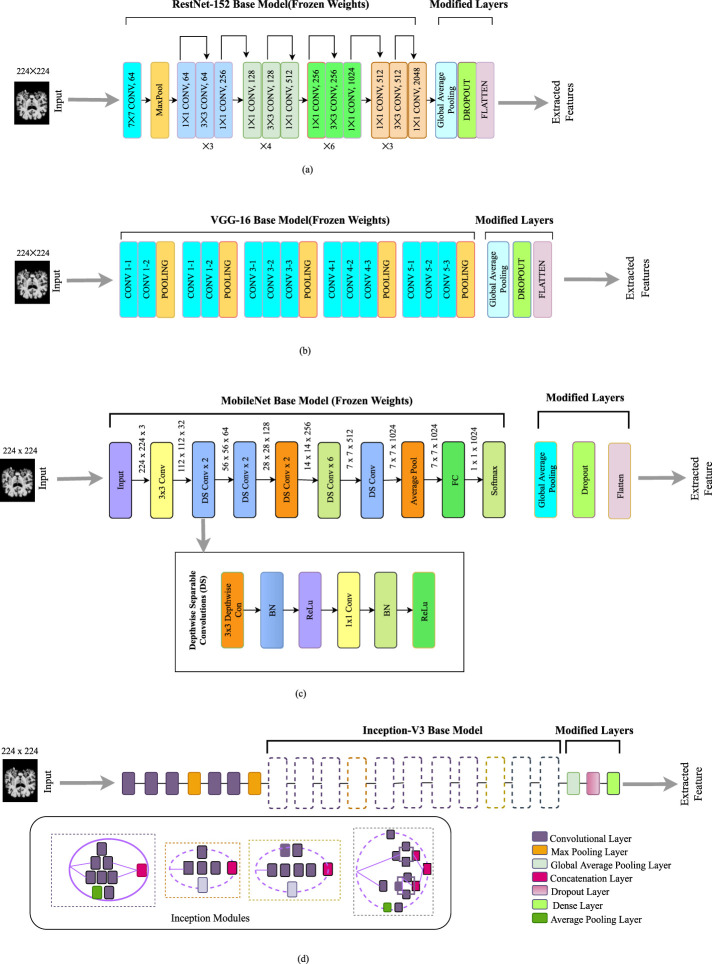
Feature extractors employed in this work: **(a)** Modified ResNet-152 model. **(b)** Modified VGG- 16 model. **(c)** Modified MobileNet model. **(d)** Modified Inception-V3 model.

Extraction of features refers to reducing large amounts of raw data and partitioning it into further manageable components. It is done so that whenever we need data, it will be easier to process. We have used different transfer learning algorithms for feature extraction. Figure 3 illustrates every feature extractor employed in this study. Transfer learning is a beneficial method for machine learning that allows us to reuse previously trained models on a particular work and apply them to additional related tasks. This approach saves time and resources by enabling us to build on top of the knowledge contained within the pre-trained model rather than starting from scratch. On the two publicly available datasets and a merged dataset mentioned above, we have used four modified pre-trained CNN models: ResNet-152 (Roy et al., 2021), VGG-16 (Antony et al., 2022), Inception-V3 (Szegedy et al., 2016), MobileNet (Zhu et al., 2021).

#### 3.3.1 Modified ResNet152V2

Training deep neural networks can be challenging. However, a new approach called residual learning can simplify the process. This approach involves restructuring the layers of the network so that they learn residual functions that reference the inputs to the layer rather than learning functions that are not related to the inputs. This makes it easier to train networks much deeper than previously used networks. As depicted in Figure 3a, it skips connections among layers. To boost the model’s overall performance, we’ve added a global average pooling layer, along with a dropout layer and a flattened layer, on top of the base layer. The purpose of these layers is to further refine the features learned by the model and to prevent overfitting during the training process.

#### 3.3.2 Modified VGG-16

The Visual Geometry Group at Oxford is in charge of developing the VGG framework, as its name suggests. It focuses on using deep convolutional layers to enhance performance (Antony et al., 2022). There are multiple versions of the VGG architecture, but for our purposes, we have fine-tuned the VGG- 16 architecture with some modifications. Feature extraction and feature categorization are two separate components in this model. The feature extraction segment is made up of a series of Convolution (Conv) layers for each Convolution (Conv) block, followed by a fuzzy layer after the pooling layer (Hossain et al., 2022). We have modified the main architecture. A dropout layer and flattened layer are added instead of fully connected layers. This modification is effective in improving the model’s performance by minimizing overfitting. The size of the input layer is 224 by 224 in the original architecture, which is similar to our input image. The modified architecture is presented in Figure 3b.

#### 3.3.3 Modified MobileNet

A lighter model called MobileNet was created to tackle the issue of a high processing cost and numerous parameters Sinha and El-Sharkawy (2019). By using much fewer parameters and processing time than other architectures, this approach is intended to be quicker and more effective. MobileNet uses depthwise separable convolutions instead of traditional convolutions, which have a calculation cost of just one-eighth that of traditional convolutions. We have implemented a modified version of MobileNet. At the top of the base model, we have included a global average pooling layer, dropout layer, and flatten layer. This architecture overall reduces the complexity of the task. The architecture of the modified MobileNet is depicted in Figure 3c.

#### 3.3.4 Modified Inception-V3

The Inception network has undergone several versions Szegedy et al. (2016), with the Inception-V3 being an improvement on the original architecture. The Inception-V3 is structured to build a deeper network using a sparsely connected framework including multiple inception modules Aurna et al. (2022). Each successive module receives input from the previous stage, which is then processed by multiple convolutional filters. The outputs from these filters are combined and passed as input to the next stage. In this particular paper, the Inception-V3 architecture is used, but with some modified layers added at the top of the base model which is depicted in Figure 3d. Furthermore, a global average pooling layer has been implemented in place of the global average pooling layer, and a dense layer has been used in place of the fully connected layer. These modifications are made using a random search approach, similar to the modified EfficientNet-B0 model.

#### 3.3.5 Convolutional neural network

Convolutional Neural networks can learn complex patterns and relationships in data, making them well-suited for a wide range of tasks, including image recognition, natural language processing, and time-series prediction. In most practical applications of neural networks, the Multilayer Perceptron (MLP) is commonly used which is typically trained using either the Backpropagation algorithm or the gradient descent method (Bangyal et al., 2011). The networks of CNN use specialized layers such as convolutional layers for feature extraction, pooling layers for dimensionality reduction, and fully connected layers for high-level reasoning. Increased neuron number enhances a convolutional neural network’s performance and efficiency, but it can also decrease its ability to generalize resulting in overfitting issues (Mumuni and Mumuni, 2022). In our work, we have carefully rationalized the size of each dataset used to ensure optimal performance and generalization. We have balanced the dataset sizes to avoid overfitting while maintaining sufficient training data for robust model learning. The architectural design of CNNs allows them to automatically learn and extract important features, making them the foundation of modern computer vision systems. In our work, we have implemented a 2D Convolutional Neural Network and carefully adjusted dataset sizes to ensure effective training and generalization.

### 3.4 Proposed model

To identify Alzheimer’s phases, we have modified ResNet- 152V2 and utilized it as both a feature extractor and a Convolutional Neural Network (CNN) classifier. We have rigorously evaluated various combinations of two classifiers and four feature extractors to determine the most effective configuration for constructing our final model, enabling reliable detection of Alzheimer’s stages. Our final model incorporates an input layer with 224 × 224 × 3 dimensions.

We have adopted a Sequential architecture in the proposed model, integrating both ResNet152V2 and InceptionV3 models for classification. The proposed model is named as IncepRes in this work. This ensemble approach leverages the strengths of both architectures to capture a more comprehensive representation of the input data. The integration of Inception modules into the ResNet architecture enhances its feature extraction capabilities, allowing for improved detection of subtle patterns indicative of Alzheimer’s stages.

Several modifications have been implemented to optimize the performance of ResNet152-V2 and InceptionV3. The figure includes layers such as 1 × 1 with Conv 64 channel, 3 × 3 convolutions with Conv 256 channel, dropout layers, and global average pooling. After feature extraction, the extracted features are passed through a fully connected Dense layer consisting of 264 units, followed by Batch Normalization to stabilize and accelerate training. Global Average Pooling layers are employed in both ResNet152V2 and InceptionV3 to reduce the number of parameters while retaining spatial information. A Dropout layer (with a rate of 0.2) is added after each block to mitigate overfitting. The output of this layer is processed through another Dense layer with 64 units for further feature refinement. The final classification layer is a Dense layer with a Softmax activation function, which provides a probability distribution over the different Alzheimer’s stages.

**FIGURE 4:**
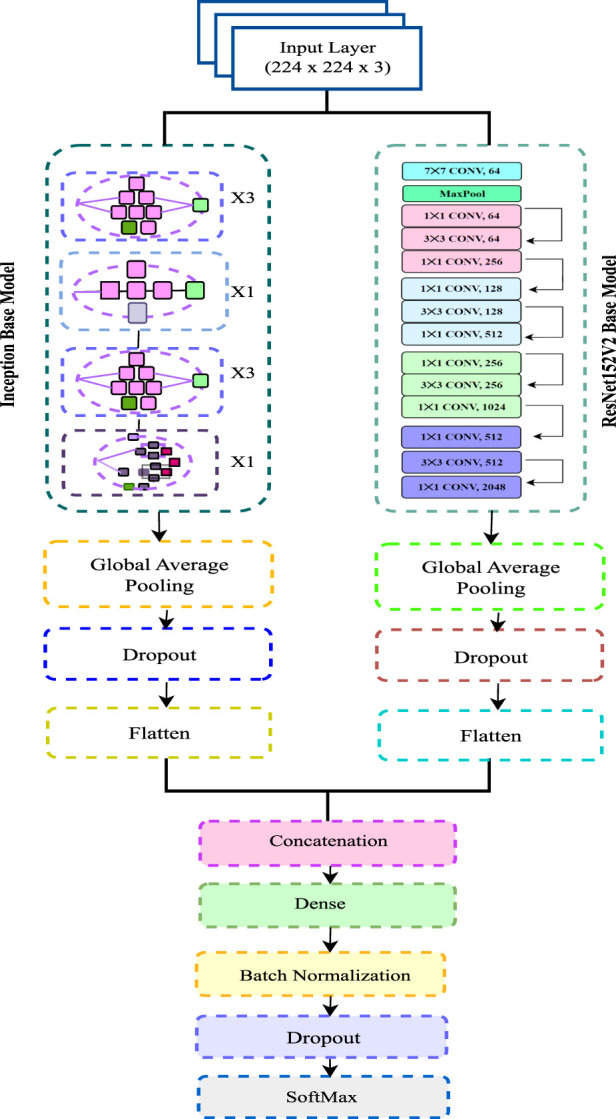
Architecture of the proposed model.

Considering the datasets used, ADNI, OASIS and merged, our proposed model aims to leverage the rich and diverse data available in these datasets to enhance the accuracy and robustness of Alzheimer’s stage detection. The architectural design of the model is illustrated in Figure 4.

### 3.5 Hyperparameter tuning

Hyperparameter tuning consists of comparing several combinations of hyperparameters to determine which produces the highest accuracy or smallest error on a validation set. This can be done manually by trying different values for each hyperparameter, or automatically using techniques such as grid search, random search, and Bayesian optimization. We have used ReLU as an input activation function. ReLU, short for Rectified Linear Unit, is a popular activation function used in deep learning that returns the input if it is positive, and 0 otherwise, and can be expressed using the mathematical formula in Equation 1:

$$f\;\left(x\right)=\max\left(0,x\right)$$ (1)

Softmax is used as the output activation function for our multi-class classification problem. The model is optimized with Adam as the optimizer which can be thought of as a fusion of AdaGrad and RMSProp. Different parameters that we have used as hyperparameters to train the model are given in Table 3.

**TABLE 3:** Hyperparameter values utilized to train the proposed model.

Hyperparameter	Value
Train-test split	90%–10%
Activation function (Input)	ReLU
Activation function (Output)	Softmax
Optimizer	Adam
Batch size	32
Epochs	30
Dropout rate	0.2
Early stopping patience	5
Initial learning rate	0.0001
Learning rate decay	0.1

### 3.6 Training and validation of model

**FIGURE 5:**
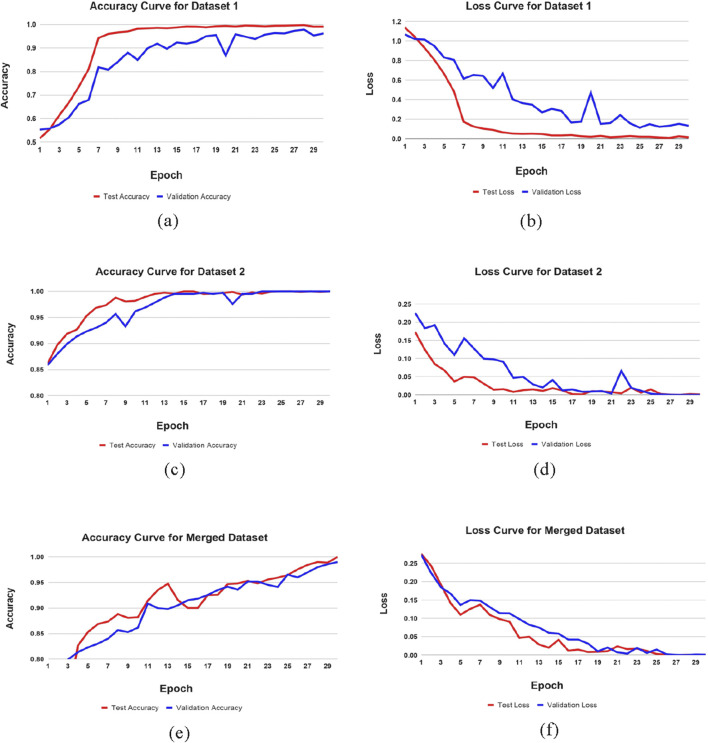
Training performance metrics across datasets. **(a)** Accuracy curve of Dataset 1, **(b)** Loss curve of Dataset 1, **(c)** Accuracy curve of Dataset 2, **(d)** Loss curve of Dataset 2, **(e)** Accuracy curve of Merged Dataset, **(f)** Loss curve of Merged Dataset.

To maximize the efficiency of detecting phases of Alzheimer’s disease (AD) detection, our research has implemented an optimal methodology. The proposed ResNet152 architecture, in combination with the convolutional neural network classifier, has specifically produced substantial gains in validation accuracy and a faster decline in validation loss. Our results indicate that ResNet-152 combined with IncepRes classifiers can be useful approaches for enhancing the accuracy and effectiveness of Alzheimer’s Phase detection. Figure 5 illustrates this statement. After 30 iterations, the learning state has reached to a nearly stable state.

## 4 Results

In this study, the efficiency of four modified transfer learning-based feature extractors in extracting features from sample data was evaluated. A performance comparison of these feature extractors is run to discover which produced the best results. In addition, our data is classified using two distinct classifiers and analyzed for their effectiveness. This research aims to classify multiple stages of AD by finding the best combination of feature extractor and classifier. The study’s findings have important implications for developing image classification systems.

### 4.1 Performance demonstration

Accuracy is an indicator that provides a basic and easy evaluation of the efficacy of a classification model. Precision is quantified in percentages, with a greater one indicating better performance. The recall is the proportion of true positive predictions made by the model to the total number of positive samples. The F-measure (F1 score) combines “precision” and “recall,” which are calculated based on the number of properly identified samples, and both false positives and false negatives, and it provides a balance between the two (Khatun et al., 2022). The performance matrix formulas are provided in Equations 2–5.

$$Accuracy=\frac{TP+TN}{TP+TN+FP+FN}$$ (2)

$$Precision=\frac{TP}{TP+FP}$$ (3)

$$\text{Recall}=\frac{TP}{TP+FN}$$ (4)

$$F1-\text{Score}=\frac{2*Precision*Recall}{Precision+Recall}=\frac{2*TP}{2\;*\;TP+FP+FN}$$ (5)

Two distinct datasets and a merged dataset are utilized to train the proposed model. Table 4 presents the findings for all performance indicators obtained from the suggested approach for the individual datasets and shows a significant improvement in performance metrics for different datasets after using our proposed model. This improvement indicates the model’s robustness and effectiveness in handling diverse data characteristics across different datasets.

**TABLE 4:** Result analysis of proposed model.

Dataset	Sample size	Accuracy	Precision	Recall	F1 score	Validation loss
Dataset 1	6,564	96.96%	97.05%	96.96%	96.96%	0.11305
Dataset 2	2,310	98.35%	98.35%	98.35%	98.35%	0.00017
Merged Dataset	8,190	97.13%	97.11%	98.00%	97.13%	0.001053

**FIGURE 6:**
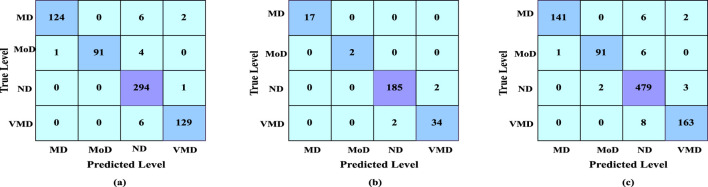
**(a)** Confusion matrix of dataset 1, **(b)** confusion matrix of dataset 2, **(c)** confusion matrix of merged dataset.

The confusion matrix illustrates the various ways in which a classification model can become confused when making assumptions. According to the count values that are broken down based on each class, the confusion matrix summarizes the amount of true and false assumptions. This matrix helps in identifying specific classes where the model performs well or struggles, providing insights into potential areas for improvement. The confusion matrix of our result is shown in Figure 6.

### 4.2 Comparison of all the combined model

**FIGURE 7:**
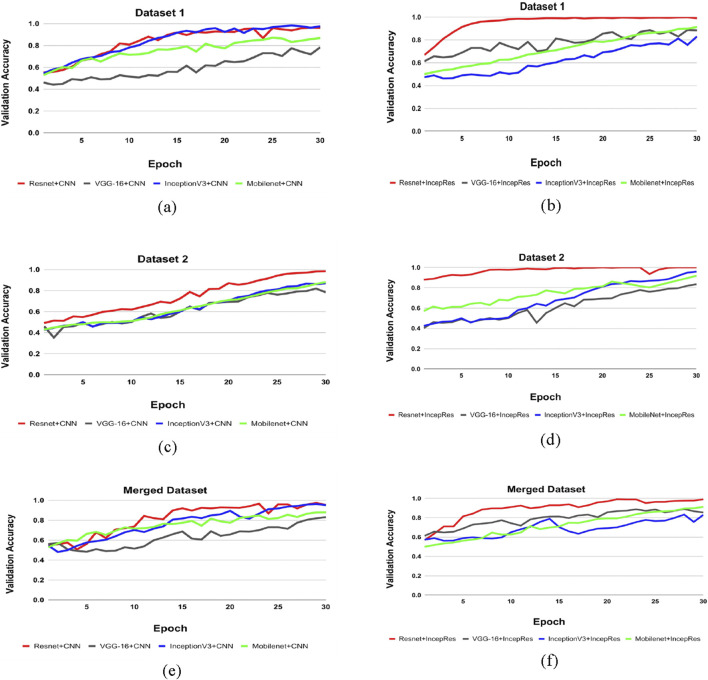
Comparison of feature extractors across datasets. **(a)** CNN for Dataset 1, **(b)** IncepRes for Dataset 1, **(c)** CNN for Dataset 2, **(d)** IncepRes for Dataset 2, **(e)** CNN for Merged Dataset, **(f)** IncepRes for Merged Dataset.

Table 5 shows the accuracy, precision, recall and f1-score of all combination models using four feature extractors along with two classifiers. The results demonstrate variability in performance across different datasets, emphasizing the importance of selecting the right feature extractor and classifier combination for optimal results. Though different combination shows better results for one dataset in terms of another dataset, we can see the difference. It can be demonstrated that our proposed model, a modified ResNet152V2 combined with IncepRes, provides the best accuracy for both the ADNI and OASIS datasets, as well as the merged dataset. In Figure 7a, the validation accuracy against epochs for four feature extractors, including convolutional neural network as a classifier for Dataset 1, can be seen visually. For the four feature extractors combined with IncepRes, the validation accuracy versus epochs graph is depicted in Figure 7b. Figure 7c provides a lucid illustration of the validation accuracy versus epoch graph for four feature extractors utilizing the convolutional neural network. Figure 7d illustrates the same for feature extractors utilizing our proposed classifier concerning Dataset 2. In all six graphs, we can see that the combination of modified Resnet152V2 with IncepRes gives us optimized results in terms of validating the sample data.

**TABLE 5:** Performance of all models.

Dataset name	Feature extractor	Classifier	Accuracy	Precision	Recall	F1 score
Dataset 1	ResNet152V2	CNN	95.57%	95.59%	95.57%	95.57%
		IncepRes	96.96%	97.05%	96.96%	96.96%
	InceptionV3	CNN	96.41%	96.47%	96.41%	96.41%
		IncepRes	76.56%	78.37%	76.56%	76.56%
	MobileNetV2	CNN	85.75%	84.65%	85.75%	85.75%
		IncepRes	90.88%	91.37%	90.88%	90.88%
	VGG-16	CNN	65.09%	66.05%	65.09%	65.09%
		IncepRes	85.76%	85.48%	85.76%	85.76%
Dataset 2	ResNet152V2	CNN	97.68%	96.08%	97.68%	97.68%
		IncepRes	98.35%	98.35%	98.35%	98.35%
	InceptionV3	CNN	85.56%	83.96%	85.56%	85.56%
		IncepRes	95.56%	95.49%	95.56%	95.56%
	MobileNetV2	CNN	86.85%	86.76%	86.85%	86.85%
		IncepRes	92.70%	92.55%	92.70%	92.70%
	VGG-16	CNN	74.13%	54.96%	74.13%	74.13%
		IncepRes	82.83%	79.98%	82.83%	82.83%
Merged Dataset	ResNet152V2	CNN	96.58%	95.07%	96.58%	96.58%
		IncepRes	97.13%	97.11%	98.00%	97.13%
	InceptionV3	CNN	87.36%	83.96%	84.76%	84.66%
		IncepRes	93.52%	94.44%	94.56%	94.16%
	MobileNetV2	CNN	86.89%	87.16%	87.15%	86.82%
		IncepRes	91.78%	91.55%	92.20%	92.30%
	VGG-16	CNN	79.30%	64.99%	74.13%	69.34%
		IncepRes	82.56%	80.98%	80.80%	81.44%

### 4.3 Effect of data augmentation

Modern machine learning models generally require a large amount of high-quality, annotated data for good performance. Often, it is not feasible to gather enough medical data to train a model for security issues. The optimal way to deal with this problem is data augmentation. Increasing the quantity, quality, and diversity of training data is the primary objective of data augmentation (Al Shehri, 2022). Six steps of data augmentation are used while training our model. From Table 6, we can see that before augmenting our data, we have got 89.23%, 89.29% and 88.31% respectively for Dataset 1, Dataset 2 and merged dataset. In another way, after applying the augmentation process, 96.96% accuracy for Dataset 1, 98.35% accuracy for Dataset 2% and 97.13% for merged dataset are obtained. So, an increase in results can be achieved for different datasets after augmenting the data.

**TABLE 6:** Accuracy before and after data augmentation.

Dataset	Before augmentation	After augmentation
Dataset 1	89.23%	96.96%
Dataset 2	89.29%	98.35%
Merged Dataset	88.31%	97.13%

### 4.4 Validating model robustness

Statistical as well as machine-learning approaches are now being utilized in high-stakes applications. To determine how reliable a method is, it is vital to carry out an assessment. The reliability of our methods can be evaluated using assessment techniques like cross-validation (CV) and robustness checks. Three experiments—training and validation using different datasets, random dataset splitting and k-fold cross-validation—are carried out to validate the adaptability and generalization capability of the proposed model.

#### 4.4.1 Training and validation using different datasets

To evaluate the generalization capability of the model, we have employed a cross-corpus approach. Using the OASIS and ADNI datasets, we have alternated between training on one and validating on the other, allowing two combinations for testing the ensemble model. The results of this process are presented in Table 7.

**TABLE 7:** Performance evaluation based on cross-corpus validation.

Training set	Validation set	Validation accuracy
Dataset 1	Dataset 2	98.11
Dataset 2	Dataset 1	95.22

#### 4.4.2 Random splitting of sample data

As shown in Table 8, we have split our datasets into varying amounts of training and test sets to assess the integrity of our model. This experiment is designed to evaluate the effects of random splitting on the functionality of our model. We can see that despite this random division, the model’s accuracy does not significantly degrade. Three ratios have been put to the test, giving the training set, respectively, 90%, 80%, and 70% of the data. Notably, regardless of the different split ratios, our model’s accuracy remains consistently competitive across Dataset 1, Dataset 2 and the Merged dataset.

**TABLE 8:** Accuracy for randomly splitting data.

Dataset	Train/Test split	Accuracy (%)	Avg accuracy (%)
Dataset 1	90%–10%	96.96	96.43
	80%–20%	96.54	
	70%–30%	95.78	
Dataset 2	90%–10%	98.34	97.63
	80%–20%	98.22	
	70%–30%	96.33	
Merged	90%–10%	97.13	96.98
	80%–20%	97.49	
	70%–30%	96.33	

#### 4.4.3 K-fold cross validation

K-fold cross-validation is a method that is frequently used in machine learning to measure a model’s performance. It implies dividing the dataset into K nearly equal folds or subsets. Then, the model is trained K times, with the remaining K-1 folds serving as training data and each of the K folds serving as validation data once. The effectiveness of the model is determined by averaging the K validation scores.

**FIGURE 8:**
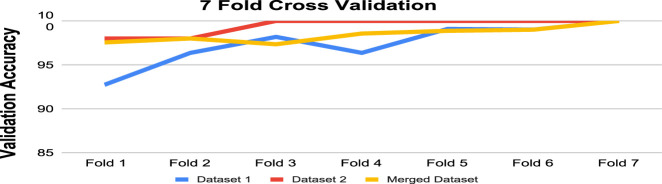
Validation accuracy after 7-fold validation for Datasets.

A model’s performance can be estimated more precisely using K-fold cross-validation than by using just one train-test split. We have used 7-fold cross-validation in this work. Table 9 and Figure 8 demonstrate the performance of 7-fold cross-validation. In addition, after collecting performance metrics for each fold, we have computed the standard deviation. The average accuracy and corresponding standard deviation across the 7 folds were as follows: 97.25% ± 2.30 for Dataset 1, 99.14% ± 0.86 for Dataset 2, and 98.19% ± 0.81 for the Merged Dataset. These results indicate that the proposed model performs consistently across different subsets of data, further supporting its robustness and generalization capability.

**TABLE 9:** Performance of K-fold cross validation.

Dataset	Fold 1	Fold 2	Fold 3	Fold 4	Fold 5	Fold 6	Fold 7
Dataset 1	92.73	96.36	98.18	96.36	99.09	99.01	100
Dataset 2	98.00	98.00	100	100	100	100	100
Merged Dataset	97.55	98.00	97.34	98.56	98.87	99.00	100

### 4.5 Comparison with other similar approaches

The suggested model is compared with other existing approaches considering various evaluation metrics. Table 10 depicts the performance of different evaluation metrics of some related existing works for Dataset 1 and Table 11 shows the results for Dataset 2. We have selected algorithms that closely align with our approach to provide better insights and a more meaningful comparison. Analyzing Table 10, we can observe that our proposed model provides better accuracy than existing models. Table 11 also shows that the performance matrices of our proposed model have outperformed other existing models.

**TABLE 10:** Performance comparison of the proposed model with other similar approaches considering dataset 1.

References	Method used	Accuracy (%)	Precision (%)	Recall (%)
Shaji et al. (2021)	Inception-ResNet	69.00	76.00	76.00
Mohi ud din dar et al. (2023)	MobileNet	96.22	95.55	96.45
Al Shehri (2022)	ResNet50	88.70	81.92	87.30
Aaraji and Abbas (2022)	3D ResNet-20	93.75	91.92	92.30
Angkoso et al. (2022)	Mp-CNN	93.00	93.00	93.00
Ghaffari et al. (2022)	InceptionV3 with TL	93.75	90.97	90.62
Proposed Model	ResNet152+IncepRes	**96.96**	**97.05**	**96.96**

**TABLE 11:** Performance comparison of the proposed model with other similar approaches considering dataset 2.

References	Method used	Accuracy (%)	Precision (%)	Recall (%)
Ghaffari et al. (2022)	InceptionV3	93.33	93.33	93.33
Chui et al. (2022)	GAN + CNN + TL	97.50	97.30	97.50
Lin and Lin (2021)	3D-CNN	97.02	94.58	92.34
Tuan et al. (2022)	CNN, XGBoost, SVM	88.00	-	-
Balasundaram et al. (2023)	CNN + XGBoost	94.00	95.65	91.66
Proposed Model	ResNet152+IncepRes	98.35	98.35	98.35

## 5 Discussion

Identification in the early stage of Alzheimer’s Disease is necessary for the diagnosis of this disease. A critical step for clinicians is the adoption of computer-aided procedures to identify Alzheimer’s disease. The multi-class classification of Alzheimer’s Disease from the input data has been successfully enhanced using the IncepRes model, a combined CNN architecture, in our work. TL has been used to extract features from Magnetic Resonance Imaging for multi-modal Alzheimer’s disease classification and identification. ResNet152V2 is selected as the optimal feature extractor from a collection of four distinct transfer learning models and IncepRes is selected as the most accurate classifier from two classifiers. The datasets have has four stages (non-demented, mild demented, very mild demented, and moderate demented). The executed model has delivered exceptional results, efficiently utilizing a small sample size of data and thereby eliminating the necessity for an extensive collection of MRI images through intelligent training data selection. ResNet152V2, along with IncepRes, provides a standard accuracy which is 96.96% for Dataset 1, 98.35% for Dataset 2% and 97.13% for merged dataset. Having conducted comprehensive experiments, the model has been validated by comparing it to existing models and evaluating its performance on multiple datasets.

Our current study has some limitations. It relies on pre-labeled data and validates on small datasets. These elements could affect the generality of the model. Noise can be introduced into MRI images by motion artifacts and due to the nature of the imaging process. Additionally, the model may face bias due to the overlapping nature and limited size of the datasets. The model should be used for bigger and more varied datasets in the future, and new imaging modalities should be included to improve diagnostic accuracy.

## 6 Conclusion

Precise identification with different AD stages is a challenging task and in this study, we present a robust framework for multi-class classification of four AD stages using MRI imaging by fusing the Inception and ResNet architectures. The proposed model outperformed the pre-trained models as well as state-of-the-art models, as ResNet152V2 demonstrated to be the most effective feature extractor among other transfer learning models. This approach not only demonstrates superior accuracy compared to existing models but also effectively utilizes limited data. In order to diagnose AD in a timely manner, early detection and precise classification of the disease must be addressed by the suggested methodology. In real-world clinical settings, where huge datasets are not readily available, this proposed model can be applied due to its ability to perform with high accuracy with limited data. Moreover, regular healthcare processes may benefit from the model’s widespread adoption, as proved by its robustness across various datasets. In future, we will consider the addition of an attention layer to the convolutional neural network architecture to help in concentrating on areas with less noise or suppress noise in specific areas. We will use an open-source platform to collect data, including video, natural language processing, and voice, all of which correspond to symptoms. Lastly, it would be intriguing to incorporate the patient’s medical history to improve the data obtained from MRIs, guide the decision-making process, and correlate it with patients’ records. We hope that our proposed model will benefit the effective screening of various AD stages and aid AD-related disability management.

## Data Availability

Publicly available datasets were analyzed in this study. This data can be found here: https://www.oasis-brains.org/and https://adni.loni.usc.edu/.

## References

[B1] Aaraji Z. S. Abbas H. H. (2022). Automatic classification of alzheimer’s disease using brain mri data and deep convolutional neural networks. arXiv preprint arXiv:2204.00068.

[B2] ADNI (2003). Data used in the preparation of this article were obtained from the Alzheimer’s Disease. Available online at: http://adni.loni.usc.edu.

[B3] Ahamed K. U. Islam M. Uddin A. Akhter A. Paul B. K. Yousuf M. A. (2021). A deep learning approach using effective preprocessing techniques to detect covid-19 from chest ct-scan and x-ray images. Comput. Biol. Med. 139, 105014. 10.1016/j.compbiomed.2021.105014 34781234 PMC8566098

[B4] AlSaeed D. Omar S. F. (2022). Brain mri analysis for alzheimer’s disease diagnosis using cnn-based feature extraction and machine learning. Sensors 22, 2911. 10.3390/s22082911 35458896 PMC9025443

[B5] Al Shehri W. (2022). Alzheimer’s disease diagnosis and classification using deep learning techniques. PeerJ Comput. Sci. 8, e1177. 10.7717/peerj-cs.1177 PMC10280208 37346304

[B6] Angkoso C. V. Agustin Tjahyaningtijas H. P. Purnama I. Purnomo M. H. (2022). Multiplane convolutional neural network (mp-cnn) for alzheimer’s disease classification. Int. J. Intelligent Eng. and Syst. 15.

[B7] Antony F. Anita H. George J. A. (2022). “Classification on alzheimer’s disease mri images with vgg-16 and vgg-19,” in IOT with smart systems: proceedings of ICTIS 2022 (Springer), 2, 199–207. 10.1007/978-981-19-3575-6_22

[B8] Arafa D. A. Moustafa H. E.-D. Ali-Eldin A. M. Ali H. A. (2022). Early detection of alzheimer’s disease based on the state-of-the-art deep learning approach: a comprehensive survey. Multimedia Tools Appl. 81, 23735–23776. 10.1007/s11042-022-11925-0

[B9] Aurna N. F. Yousuf M. A. Taher K. A. Azad A. Moni M. A. (2022). A classification of mri brain tumor based on two stage feature level ensemble of deep cnn models. Comput. Biol. Med. 146, 105539. 10.1016/j.compbiomed.2022.105539 35483227

[B10] Bae J. B. Lee S. Jung W. Park S. Kim W. Oh H. (2020). Identification of alzheimer’s disease using a convolutional neural network model based on t1-weighted magnetic resonance imaging. Sci. Rep. 10, 22252. 10.1038/s41598-020-79243-9 33335244 PMC7746752

[B11] Balasundaram A. Srinivasan S. Prasad A. Malik J. Kumar A. (2023). Hippocampus segmentation-based alzheimer’s disease diagnosis and classification of mri images. Arabian J. Sci. Eng. 48, 10249–10265. 10.1007/s13369-022-07538-2 PMC9810248 36619218

[B12] Bangyal W. H. Ahmad J. Rauf H. T. (2019). Optimization of neural network using improved bat algorithm for data classification. J. Med. Imaging Health Inf. 9, 670–681. 10.1166/jmihi.2019.2654

[B13] Bangyal W. H. Ahmad J. Shafi I. Abbas Q. (2011). “Forward only counter propagation network for balance scale weight and distance classification task,” in 2011 third world congress on nature and biologically inspired computing (IEEE), 342–347.

[B14] Bangyal W. H. Rehman N. U. Nawaz A. Nisar K. Ibrahim A. A. A. Shakir R. (2022). Constructing domain ontology for alzheimer disease using deep learning based approach. Electronics 11, 1890. 10.3390/electronics11121890

[B15] Bangyal W. H. Shakir R. Ashraf A. Qayyum Z. U. Rehman N. U. (2023). “Automatic detection of diabetic retinopathy from fundus images using machine learning based approaches,” in 2023 25th international multitopic conference (INMIC) (IEEE), 1–6.

[B16] Bi X. Li S. Xiao B. Li Y. Wang G. Ma X. (2020). Computer aided alzheimer’s disease diagnosis by an unsupervised deep learning technology. Neurocomputing 392, 296–304. 10.1016/j.neucom.2018.11.111

[B17] Bucholc M. Titarenko S. Ding X. Canavan C. Chen T. (2023). A hybrid machine learning approach for prediction of conversion from mild cognitive impairment to dementia. Expert Syst. Appl. 217, 119541. 10.1016/j.eswa.2023.119541

[B18] Chui K. T. Gupta B. B. Alhalabi W. Alzahrani F. S. (2022). An mri scans-based alzheimer’s disease detection via convolutional neural network and transfer learning. Diagnostics 12, 1531. 10.3390/diagnostics12071531 35885437 PMC9318866

[B19] Ebrahimi A. Luo S. Chiong R. Alzheimer's Disease Neuroimaging Initiative (2021a). Deep sequence modelling for alzheimer’s disease detection using mri. Comput. Biol. Med. 134, 104537. 10.1016/j.compbiomed.2021.104537 34118752

[B20] Ebrahimi A. Luo S. Disease Neuroimaging Initiative f. t. A. (2021b). Convolutional neural networks for alzheimer’s disease detection on mri images. J. Med. Imaging 8, 024503. 10.1117/1.jmi.8.2.024503 PMC8083897 33937437

[B21] El-Assy A. Amer H. M. Ibrahim H. Mohamed M. (2024). A novel cnn architecture for accurate early detection and classification of alzheimer’s disease using mri data. Sci. Rep. 14, 3463. 10.1038/s41598-024-53733-6 38342924 PMC10859371

[B22] Faisal F. U. R. Kwon G. R. (2022). Automated detection of alzheimer’s disease and mild cognitive impairment using whole brain mri. IEEE Access 10, 65055–65066. 10.1109/access.2022.3180073

[B23] Faruqui N. Yousuf M. A. Whaiduzzaman M. Azad A. Barros A. Moni M. A. (2021). Lungnet: a hybrid deep-cnn model for lung cancer diagnosis using ct and wearable sensor-based medical iot data. Comput. Biol. Med. 139, 104961. 10.1016/j.compbiomed.2021.104961 34741906

[B24] Ghaffari H. Tavakoli H. Pirzad Jahromi G. (2022). Deep transfer learning–based fully automated detection and classification of alzheimer’s disease on brain mri. Br. J. radiology 95, 20211253. 10.1259/bjr.20211253 PMC10162060 35616643

[B25] Ghosh T. Palash M. I. A. Yousuf M. A. Hamid M. A. Monowar M. M. Alassafi M. O. (2023). A robust distributed deep learning approach to detect alzheimer’s disease from mri images. Mathematics 11, 2633. 10.3390/math11122633

[B26] Hazarika R. A. Kandar D. Maji A. K. (2022). An experimental analysis of different deep learning based models for alzheimer’s disease classification using brain magnetic resonance images. J. King Saud University-Computer Inf. Sci. 34, 8576–8598. 10.1016/j.jksuci.2021.09.003

[B27] Helaly H. A. Badawy M. Haikal A. Y. (2022). Deep learning approach for early detection of alzheimer’s disease. Cogn. Comput. 14, 1711–1727. 10.1007/s12559-021-09946-2 PMC8563360 34745371

[B28] Hon M. Khan N. M. (2017). “Towards alzheimer’s disease classification through transfer learning,” in 2017 IEEE International conference on bioinformatics and biomedicine (BIBM) (IEEE), 1166–1169.

[B29] Hossain M. M. Hasan M. M. Rahim M. A. Rahman M. M. Yousuf M. A. Al-Ashhab S. (2022). Particle swarm optimized fuzzy cnn with quantitative feature fusion for ultrasound image quality identification. IEEE J. Transl. Eng. Health Med. 10, 1–12. 10.1109/jtehm.2022.3197923 PMC9550163 36226132

[B30] Kang W. Lin L. Zhang B. Shen X. Wu S. Initiative A. D. N. (2021). Multi-model and multi-slice ensemble learning architecture based on 2d convolutional neural networks for alzheimer’s disease diagnosis. Comput. Biol. Med. 136, 104678. 10.1016/j.compbiomed.2021.104678 34329864

[B31] Khan A. Zubair S. (2022). An improved multi-modal based machine learning approach for the prognosis of alzheimer’s disease. J. King Saud University-Computer Inf. Sci. 34, 2688–2706. 10.1016/j.jksuci.2020.04.004

[B32] Khatun M. A. Yousuf M. A. Ahmed S. Uddin M. Z. Alyami S. A. Al-Ashhab S. (2022). Deep cnn-lstm with self-attention model for human activity recognition using wearable sensor. IEEE J. Transl. Eng. Health Med. 10, 1–16. 10.1109/jtehm.2022.3177710 PMC9252338 35795873

[B33] Koga S. Ikeda A. Dickson D. W. (2022). Deep learning-based model for diagnosing alzheimer’s disease and tauopathies. Neuropathology Appl. Neurobiol. 48, e12759. 10.1111/nan.12759 PMC9293025 34402107

[B34] Lin C.-J. Lin C.-W. (2021). Using three-dimensional convolutional neural networks for alzheimer’s disease diagnosis. Sensors and Mater. 33, 3399. 10.18494/sam.2021.3512

[B35] Marcus D. S. Wang T. H. Parker J. Csernansky J. G. Morris J. C. Buckner R. L. (2007). Open access series of imaging studies (oasis): cross-sectional mri data in young, middle aged, nondemented, and demented older adults. J. Cogn. Neurosci. 19, 1498–1507. 10.1162/jocn.2007.19.9.1498 17714011

[B36] Mohi ud din dar G. Bhagat A. Ansarullah S. I. Othman M. T. B. Hamid Y. Alkahtani H. K. (2023). A novel framework for classification of different alzheimer’s disease stages using cnn model. Electronics 12, 469. 10.3390/electronics12020469

[B37] Mumuni A. Mumuni F. (2022). Data augmentation: a comprehensive survey of modern approaches. Array 16, 100258. 10.1016/j.array.2022.100258

[B38] Nagarathna C. Kusuma M. (2021). “Comparative study of detection and classification of alzheimer’s disease using hybrid model and cnn,” in 2021 international conference on disruptive technologies for multi-disciplinary research and applications (CENTCON) (IEEE), 1, 43–46.

[B39] Nawaz A. Anwar S. M. Liaqat R. Iqbal J. Bagci U. Majid M. (2020). “Deep convolutional neural network based classification of alzheimer’s disease using mri data,” in 2020 IEEE 23rd international multitopic conference (INMIC) (IEEE), 1–6.

[B40] Rajeswari S. S. Nair M. (2021). “A transfer learning approach for predicting alzheimer’s disease,” in 2021 4th biennial international conference on nascent technologies in engineering (ICNTE) (IEEE), 1–5.

[B41] Raza N. Naseer A. Tamoor M. Zafar K. (2023). Alzheimer disease classification through transfer learning approach. Diagnostics 13, 801. 10.3390/diagnostics13040801 36832292 PMC9955379

[B42] Roy P. Chisty M. M. O. Fattah H. A. (2021). “Alzheimer’s disease diagnosis from mri images using resnet-152 neural network architecture,” in 2021 5th international conference on electrical information and communication technology (EICT) (IEEE), 1–6.

[B43] Salehi A. W. Baglat P. Sharma B. B. Gupta G. Upadhya A. (2020). “A cnn model: earlier diagnosis and classification of alzheimer disease using mri,” in 2020 international conference on smart electronics and communication (ICOSEC) (IEEE), 156–161.

[B44] Shaji S. Ganapathy N. Swaminathan R. (2021). Classification of alzheimer condition using mr brain images and inception-residual network model. Curr. Dir. Biomed. Eng. 7, 763–766. 10.1515/cdbme-2021-2195

[B45] Shamrat F. J. M. Akter S. Azam S. Karim A. Ghosh P. Tasnim Z. (2023). Alzheimernet: an effective deep learning based proposition for alzheimer’s disease stages classification from functional brain changes in magnetic resonance images. IEEE Access 11, 16376–16395. 10.1109/access.2023.3244952

[B46] Sinha D. El-Sharkawy M. (2019). “Thin mobilenet: an enhanced mobilenet architecture,” in 2019 IEEE 10th annual ubiquitous computing, electronics and mobile communication conference (UEMCON) (IEEE), 0280–0285.

[B47] Sudharsan M. Thailambal G. (2023). Alzheimer’s disease prediction using machine learning techniques and principal component analysis (pca). Mater. Today Proc. 81, 182–190. 10.1016/j.matpr.2021.03.061

[B48] Szegedy C. Vanhoucke V. Ioffe S. Shlens J. Wojna Z. (2016). “Rethinking the inception architecture for computer vision,” in Proceedings of the IEEE conference on computer vision and pattern recognition, 2818–2826.

[B49] Tanveer M. Rashid A. H. Ganaie M. Reza M. Razzak I. Hua K.-L. (2021). Classification of alzheimer’s disease using ensemble of deep neural networks trained through transfer learning. IEEE J. Biomed. Health Inf. 26, 1453–1463. 10.1109/jbhi.2021.3083274 34033550

[B50] Tuan T. A. Pham T. B. Kim J. Y. Tavares J. M. R. (2022). Alzheimer’s diagnosis using deep learning in segmenting and classifying 3d brain mr images. Int. J. Neurosci. 132, 689–698. 10.1080/00207454.2020.1835900 33045895

[B51] Ullanat V. Balamurali V. Rao A. (2021). “A novel residual 3-d convolutional network for alzheimer’s disease diagnosis based on raw mri scans,” in 2020 IEEE-EMBS conference on biomedical engineering and sciences (IECBES) (IEEE), 82–87.

[B52] Zhang X. Han L. Zhu W. Sun L. Zhang D. (2021). An explainable 3d residual self-attention deep neural network for joint atrophy localization and alzheimer’s disease diagnosis using structural mri. IEEE J. Biomed. health Inf. 26, 5289–5297. 10.1109/jbhi.2021.3066832 33735087

[B53] Zhu Y. Liang X. Batsis J. A. Roth R. M. (2021). Exploring deep transfer learning techniques for alzheimer’s dementia detection. Front. Comput. Sci. 3, 624683. 10.3389/fcomp.2021.624683 34046588 PMC8153512

